# Chemical and Colloidal Stability of Polymer-Coated NaYF_4_:Yb,Er Nanoparticles in Aqueous Media and Viability of Cells: The Effect of a Protective Coating

**DOI:** 10.3390/ijms24032724

**Published:** 2023-02-01

**Authors:** Mykhailo Nahorniak, Vitalii Patsula, Dana Mareková, Petr Matouš, Oleksandr Shapoval, Viktoriia Oleksa, Magda Vosmanská, Lucia Machová Urdzíková, Pavla Jendelová, Vít Herynek, Daniel Horák

**Affiliations:** 1 Institute of Macromolecular Chemistry, Czech Academy of Sciences, Heyrovského nám. 2, 160 00 Prague, Czech Republic; 2 Institute of Experimental Medicine, Czech Academy of Sciences, Vídeňská 1083, 142 20 Prague, Czech Republic; 3 Department of Neurosciences, Second Faculty of Medicine, Charles University, V Úvalu 84, 150 06 Prague, Czech Republic; 4 Center for Advanced Preclinical Imaging, First Faculty of Medicine, Charles University, Salmovská 3, 120 00 Prague, Czech Republic; 5 Department of Analytical Chemistry, University of Chemistry and Technology Prague, Technická 5, Prague 6, 166 28 Prague, Czech Republic

**Keywords:** luminescence, upconversion, nanoparticles, lanthanides, degradation

## Abstract

Upconverting nanoparticles (UCNPs) are of particular interest in nanomedicine for in vivo deep-tissue optical cancer bioimaging due to their efficient cellular uptake dependent on polymer coating. In this study, particles, *ca.* 25 nm in diameter, were prepared by a high-temperature coprecipitation of lanthanide chlorides. To ensure optimal dispersion of UCNPs in aqueous milieu, they were coated with three different polymers containing reactive groups, i.e., poly(ethylene glycol)-alendronate (PEG-Ale), poly(*N*,*N*-dimethylacrylamide-*co*-2-aminoethylacrylamide)-alendronate (PDMA-Ale), and poly(methyl vinyl ether-*co*-maleic acid) (PMVEMA). All the particles were characterized by TEM, DLS, FTIR, and spectrofluorometer to determine the morphology, hydrodynamic size and ξ-potential, composition, and upconversion luminescence. The degradability/dissolution of UCNPs in water, PBS, DMEM, or artificial lysosomal fluid (ALF) was evaluated using an ion-selective electrochemical method and UV-Vis spectroscopy. The dissolution that was more pronounced in PBS at elevated temperatures was decelerated by polymer coatings. The dissolution in DMEM was relatively small, but much more pronounced in ALF. PMVEMA with multiple anchoring groups provided better protection against particle dissolution in PBS than PEG-Ale and PDMA-Ale polymers containing only one reactive group. However, the cytotoxicity of the particles depended not only on their ability to rapidly degrade, but also on the type of coating. According to MTT, neat UCNPs and UCNP@PMVEMA were toxic for both rat cells (C6) and rat mesenchymal stem cells (rMSCs), which was in contrast to the UCNP@Ale-PDMA particles that were biocompatible. On the other hand, both the cytotoxicity and uptake of the UCNP@Ale-PEG particles by C6 and rMSCs were low, according to MTT assay and ICP-MS, respectively. This was confirmed by a confocal microscopy, where the neat UCNPs were preferentially internalized by both cell types, followed by the UCNP@PMVEMA, UCNP@Ale-PDMA, and UCNP@Ale-PEG particles. This study provides guidance for the selection of a suitable nanoparticle coating with respect to future biomedical applications where specific behaviors (extracellular deposition vs. cell internalization) are expected.

## 1. Introduction

In the last decade, luminescent lanthanide-doped upconverting nanoparticles (UCNPs) have gained considerable attention as a promising theranostic agent in biome-dicine [1,2]. This interest was triggered by their fascinating optical properties, mainly due to the conversion of near-infrared (NIR) irradiation to high-energy visible or ultraviolet light via the anti-Stokes process [3]. This allowed the local delivery of high-energy irradiation deep into biological tissues with low autofluorescence, long luminescent lifetime, and strong photochemical stability of the light mediator. As a result, the UCNPs proved to be a versatile tool for bioimaging, sensing, and therapeutical applications, including food assays and hazard detection [4], medical diagnostics [5], or photodynamic therapy (PDT) [6]. PDT consists in the induction of apoptosis or necrosis in tissue (especially tumor tissue) by reactive oxygen species produced by the interaction of visible light emitted by particles with bound suitable dyes (e.g., phthalocyanines, chlorines, porphyrins, riboflavin, Rose Bengal, methylene blue) after irradiation with near-infrared light. Infrared light easily penetrates the tissue and therefore allows excitation of particles deep in the body, whereas visible light has only a local effect. The surrounding tissue is therefore not at risk. Many different materials are used for deep-penetrating PDT, such as metal or carbon nanoparticles, semiconductor quantum dots, fluorescent dyes, or lanthanide-doped UCNPs. In the latter, the two most commonly used rare-earth ion pairs are ytterbium-erbium or ytterbium-thulium. 

In biomedical applications, UCNPs have to be well-dispersible in aqueous media, chemically and colloidally stable, and provide a high luminescence signal reproducibility [7]. These criteria depend on the properties of particles such as uniformity and phase purity that are controlled by the synthesis parameters. A variety of methods have been developed for the fabrication of rare-earth-doped UCNPs, including high-temperature coprecipitation or decomposition, microemulsion, microwave-assisted or hydrothermal synthesis [8,9,10,11]. In particular, the first approach is considered to be simple and conve-nient for the synthesis of uniform and crystalline UCNPs of different sizes and composition [10]; typically, the particles are stabilized by oleic acid. Another crucial aspect of UCNPs is their biocompatibility and nontoxicity depending on the particle size, composition, and surface chemistry [12]. Recent studies have shown that fluoride-based UCNPs have a tendency to dissolve in phosphate buffers due to the leakage of rare-earth and F^−^ ions [13,14]. This process induced a time-dependent decrease in luminescence intensity, significant heterogeneity in particle brightness, and could induce cell death [15,16,17]. The leaching of Y^3+^ ions could affect genotoxicity and/or cytotoxicity, and promote neuronal cell death [18,19]. 

Although many reports on the preparation and application of UCNPs are published annually, studies dealing with the protection of particles against dissolution in aqueous media are rather scarce [20]. Therefore, the surface coating is of great importance not only to render colloidal stability to the particles in aqueous media and avoid water-related quenching of luminescence, but also to prevent the dissolution of UCNPs thereby minimizing toxicity concerns [12,21,22,23]. Moreover, the coating often provides reactive functional groups for the prospective attachment of biomolecules, drugs, photosensitizers, targeting peptides, antibodies, etc. These functionalities are exemplified by phosphates, bisphosphonates, carboxyl, sulfonate or amino groups that can replace the oleic acid on the surface of UCNPs; photolabile o-nitrobenzyl groups were also introduced [24]. Several strategies of surface UCNP engineering have been suggested, such as ligand exchange, layer-by-layer assembly, amphiphilic polymer or silica coating, etc. In this manner, diverse polymers were introduced on the particle surface, including poly(ethylene glycol) (PEG) and its derivatives [25], poly(*N*-vinylpyrrolidone) [26], polyethylenimine [27], poly(acrylic acid) [28], poly(maleic anhydride-*alt*-1-octadecene) [29], poly(D,L-lactic-*co*-glycolic acid) [30], chitosan [31], etc. The coating was optionally formed by the polymerization of monomers (glycidyl or 2-hydroxyethyl methacrylate, oligo(ethylene glycol) methyl ether methacrylate) on the UCNP surface using the “grafting-from” strategy and/or microemulsion technique [32,33]. It should also be noted that the coating of UCNPs plays a significant role in their blood circulation time and/or the particle internalization in cells. 

This report focuses on the investigation of the effect of particle coating, medium, and temperature on dissolution and cytotoxicity of NaYF_4_:Yb,Er nanoparticles (UCNPs) prepared by high-temperature coprecipitation and coated with three different polymers including poly(ethylene glycol) (PEG), poly(*N*,*N*-dimethylacrylamide-*co*-2-aminoethylacrylamide) [P(DMA-AEA)] terminated with an alendronate anchoring group, and poly(methyl vinyl ether-*co*-maleic acid) (PMVEMA). 

## 2. Results and Discussion

As biomedical applications require water-dispersible particles, the surface of the UCNPs has to be hydrophilized, typically by polymer coatings, to maintain colloidal and chemical stability and to decrease disintegration in the complex biological media. In this report, earlier prepared poly(ethylene glycol)-alendronate (PEG-Ale), poly(*N*,*N*-dimethylacrylamide-*co*-2-aminoethylacrylamide)-alendronate [(P(DMA-AEA)-Ale)], and commercial poly(methyl vinyl ether-*co*-maleic acid) (PMVEMA) were selected as protective hydrophilic coatings of the UCNPs.

P(DMA-AEA)-Ale was synthetized by a multistep reaction, starting from the RAFT copolymerization of DMA and AEC-Boc (95/5 mol/mol). According to ^1^H NMR, the polymerization was terminated at 85% conversion by exposure to air and quenching in ice; the content of AEC-Boc in the copolymer amounted to 5 mol.%, which corresponded to the starting monomer feed ratio (Supporting Information SI, Figure S1). P(DMA-AEC-Boc) had *M*_n_ = 11 kg/mol and a narrow molar mass distribution (*M*_w_/*M*_n_ =1.06). For the next stage, the anchoring alendronate group was introduced in the copolymer via carbodiimide chemistry. The presence of Ale in the polymer and the removal of *t*-Boc-protecting groups were confirmed by ^31^P and ^1^H NMR spectroscopy (δ = 18.1 ppm; Figure S2), and by the disappearance of signal “d” corresponding to methyl protons in *t*-Boc group, respectively (Figure S1).

### 2.1. Polymer-Modified UCNPs

**Figure 1:**
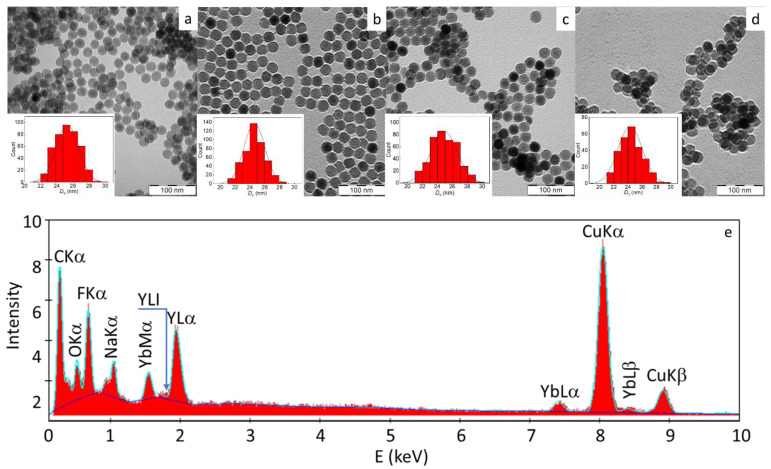
TEM micrographs of (**a**) UCNPs, (**b**) UCNP@Ale-PEG, (**c**) UCNP@Ale-PDMA, (**d**) UCNP@PMVEMA particles, and (**e**) TEM/EDX analysis of UCNPs.

The UCNPs were prepared by a high-temperature coprecipitation approach from rare-earth chlorides in a mixture of high-boiling solvent (OD) and stabilizer (OA) at 300 °C. The particles were hydrophobic due to the presence of OA stabilizer on the surface. According to TEM, the UCNPs were spherical in shape and uniform in size with *D*_n_ = 25 nm and *Ɖ* = 1.01 (Figure 1a and Table 1). The TEM/EDX spectra of UCNP exhibited major peaks at ~0.68, 1.04, and 1.91 keV corresponding to F, Na, and Y atoms, respectively (Figure 1e). Peaks at 0.23 and 8.04 keV were attributed to C and Cu atoms, respectively, originating from the supporting TEM grid. Peaks of minor intensity detected at 1.54, 7.4, and 8.42 keV confirmed the presence of Yb in the particles. The peak corresponding to Er atoms at ~6.9 keV was almost invisible due to their low concentration.

**Figure 2:**
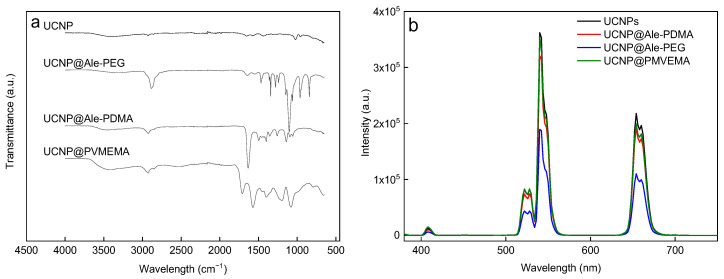
(**a**) ATR FTIR spectra and (**b**) upconversion photoluminescence emission spectra of uncoated and polymer-coated UCNPs (4 mg/mL); 980-nm excitation and laser power density 2.11 W/cm^2^.

Prior to the modification with polymers, the UCNPs were thoroughly washed with hexane, ethanol, and water to remove organic compounds (OA and OD) from the surface. The ATR FTIR spectra of the UCNPs (Figure 2a) exhibited very weak bands at 2922 and 1648 cm^−1^ attributed to asymmetric ν_as_(CH_2_) and ν(C=C) stretching vibrations [34]. Moreover, the two bands at 1574 and 1436 cm^−1^ were assigned to asymmetric and symmetric stretching vibrations of the ν(COO^−^) group, respectively, originating from the residual OA remaining after washing [35]. After the modification of the UCNPs with P(DMA-AEA)-Ale, the ATR FTIR spectrum of UCNP@Ale-PDMA exhibited a broad absorption band at 3450 cm^−1^ attributed to the stretching vibration of ν(NH) and ν(OH) originating from the amide groups and adsorbed water, respectively (Figure 2a). The peaks at 2925 and 2852 cm^−1^ were assigned to ν_as_(CH_3_) asymmetric and ν_s_(CH_2_) symmetric stretching vibrations, respectively, while the band at 1634 cm^−1^ was associated with ν(C=O) stretching vibration of the amide group, which agreed with the literature data [36,37]. After the modification of the UCNPs with PEG-Ale, the band at 3300 cm^−1^ in the ATR FTIR spectrum of UCNP@Ale-PEG was attributed to ν(OH) vibrations of adsorbed water. Moreover, the peaks at 2880 and 1102 cm^−1^ were assigned to ν_s_(CH_2_) and ν_s_(-O-) symmetric stretching vibrations of PEG, respectively [38]. The spectra of UCNP@PVMEMA nanoparticles exhibited bands at 3415, 2930, and 2850 cm^−1^ attributed to ν(OH), ν_as_(CH_3_) asymmetric and ν_s_(CH_2_) symmetric stretching vibrations, respectively. Two peaks at 1709 and 1570 cm^−1^ were assigned to ν(C=O) vibrations of COOH and COO^−^ groups, respectively. The band at 1079 cm^−1^ was attributed to ν_s_(-O-) symmetric stretching vibration of OCH_3_ groups [39]. Hence, the ATR FTIR spectroscopy confirmed the presence of PDMA, PEG, and PMVEMA on the UCNP surface.

When the UCNPs were coated with polymers, the *D*_n_ or *Ð* almost did not change (Figure 1b–d; Table 1). According to TGA, the neat UCNPs still contained 2.2 wt.% of organic coatings (OA; Table 1). The weight loss of the polymer-coated UCNPs at 120–500 °C was attributed to the polymer decomposition and indicated that the particles contained 10.3, 7.2, and 31.5 wt.% of PEG-Ale, PDMA-Ale, and PMVEMA, respectively. The hydrodynamic diameter (*D*_h_) of the neat UCNPs in water was ~100 nm (Table 1) and the ζ-potential was positive (~30 mV), probably due to the dissolution/dissociation of surface cations. *D*_h_ of UCNP@Ale-PEG, UCNP@Ale-PDMA, and UCNP@PMVEMA in water amounted to 68, 102, and 112 nm, respectively (Table 1). The increase in *D*_h_ of the polymer-coated UCNPs correlated with the increase in polymer molar mass (5, 11, and 60 kg/mol for PEG, PDMA, and PMVEMA, respectively), which can be used to control particle size. The size of the PEGylated particles was smaller than that of the uncoated ones, as a result of good stabilizing efficiency of PEG preventing aggregation. Polydispersity (*PD*) of all polymer-coated UCNPs was <0.2, confirming a narrow size distribution. In contrast to the ζ-potential of the neat UCNPs, the ζ-potential of UCNP@Ale-PEG decreased to 8 mV due to the shielding of surface charges by highly hydrophilic electroneutral PEG [38]. The UCNP@Ale-PDMA particles had *D*_h_ comparable to that of the neat UCNPs and positive ζ-potential (27 mV) due to the presence of primary amino groups on the surface. Among all the tested nanoparticles, the PMVEMA coating yielded the particles with the largest hydrodynamic diameter (112 nm), probably due to the relatively high molar mass of PMVEMA (60 kg/mol), along with its anionic nature providing a stretched conformation. Ionized carboxyl groups of PMVEMA were also responsible for the negative ζ-potential of UCNP@PMVEMA (−32 mV).

### 2.2. Upconversion (UC) Luminescence

The upconversion photoluminescence emission spectra of aqueous uncoated and polymer-coated UCNP dispersions excited at 980 nm exhibited bands at 409, 525, 542, and 656 nm corresponding to ^2^H_11/2_ → ^2^I_15/2_, ^4^S_3/2_ → ^2^I_15/2_, ^2^H_9/2_ → ^4^I_15/2_, and ^4^F_9/2_ → ^2^I_15/2_ transitions of characteristic Er^3+^ emission, respectively (Figure 2b). The modification of the UCNPs with the polymers slightly decreased the emission intensity, especially with the UCNP@Ale-PEG and UCNP@Ale-PDMA particles, probably due to irregularities in polymer grafting that resulted in water penetration and surface quenching [27]. In addition, the reduced luminescence intensity of the UCNP@Ale-PEG particles was due to their relatively small hydrodynamic diameter (*D*_h_ = 68 nm).

### 2.3. Colloidal Stability of UCNPs

The effect of several aqueous media and temperature on the colloidal stability of the neat and surface-modified UCNPs was evaluated by measuring their *D*_h_ and ζ-potential in water, PBS, and DMEM. All polymer-coated UCNPs were colloidally stable in water at 25 and 37 °C for 168 h without significant fluctuations of their hydrodynamic diameters and ζ-potentials (Figures S3a,b and S4a,b). *D*_h_ of the UCNP@Ale-PEG and UCNP@Ale-PDMA particles in PBS rapidly increased after 24 and 78 h of incubation at 25 °C, respectively; at 37 °C, *D*_h_ increased after the incubation for 6 and 24 h (Figure S3c,d), which indicated particle aggregation. This aggregation was explained by the replacement of polymer coatings with phosphates of PBS, which is in accordance with the earlier reported long-term stability vs. instability of UCNP@PEG-phosphate dispersions in water or PBS, respectively [40]. At the same time, the ζ-potential of the UCNP@Ale-PEG and UCNP@Ale-PDMA particles was approaching zero due to the formation of the counterion layer (Figure S4c,d). In contrast, the UCNP@PMVEMA particles were colloidally stable in PBS for a week, regardless of the incubation temperature and their ζ-potential remained negative (*ca*. −25 mV) for the whole period. Moreover, the neat UCNPs, UCNP@Ale-PEG, and UCNP@PMVEMA particles were well-stable in DMEM at 37 °C for 168 h without any change of *D*_h_ or *Ð* (Figure S5a,b). However, both these values for the UCNP@Ale-PDMA particles in DMEM increased due to aggregation resulting from the interaction of proteins with positively charged particles.

### 2.4. Degradability of UCNPs

**Figure 3:**
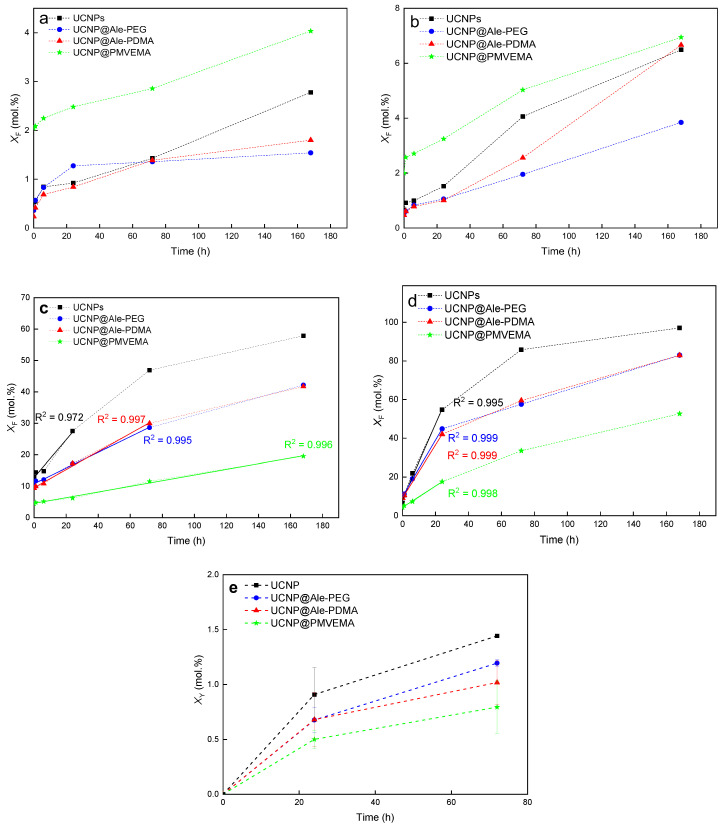
Time dependencies of (**a**–**d**) F^−^ and (**e**) Y^3+^ ion molar fraction (*X*_F_ and *X*_Y_, respectively) in supernatants. Differently coated UCNPs (1 mg/mL) were stored in (**a**,**b**) water and (**c**–**e**) PBS (pH 7.4) at (**a**,**c**) 25 and (**b**,**d**,**e**) 37 °C.

The dissolution of the neat, Ale-PEG-, Ale-PDMA-, and PMVEMA-coated UCNPs was evaluated in water and PBS at 25 and 37° by measuring F^−^ ion concentration in supernatants at different time points using a fluoride ion-selective electrode. The F^−^ ion leakage was determined as a molar fraction of dissolved F^−^ (*X*_F_) relative to the total amount of fluorine in the NaY_0.78_Yb_0.20_Er_0.02_F_4_ nanoparticles (Figure 3). To compare the ability of the coating polymers to inhibit the disintegration of the UCNPs, their dissolution rate was calculated from the linear dependence of F^−^ ion molar fraction in the supernatants on contact time (Figure 3). The dissolution curves of differently coated UCNPs indicated a noticeable increase in *X*_F_ at higher temperatures (Figure 3b,d), as a result of increased NaYF_4_ solubility [41,42]. The dissolution of the UCNPs in water, in contrast to PBS, was low (Figure 3a,b), as the mechanism of fluoride leakage is mostly based on the hydrolysis of particle surface after the exchange of all F^−^ ions for OH^−^ ones. In the case of the UCNP@PMVEMA particles, they dissolved in water more than the neat UCNPs, probably due to the complexation of PMVEMA with lanthanoids, as observed earlier with other polymers [43]. On the other hand, the disintegration of the hydrolyzed UCNPs in PBS was supported by the well-known reaction of phosphates with lanthanoids [41]. For example, F^−^ leakage from the neat UCNPs in PBS at 25 °C reached 20 mol.% after 12 h and the same degree of dissolution was observed at 37 °C already after 6 h. Alternatively, the UCNP@Ale-PEG, UCNP@Ale-PDMA, and UCNP@PMVEMA particles were more stable; they reached the same leakage (*X*_F_ = 20 mol.%) after 35-, 34-, and 168-h incubation in PBS at 25 °C, respectively (Figure 3c). The same leakage was also observed for the Ale-PEG-, Ale-PDMA-, and PMVEMA-coated UCNPs incubated at 37 °C for 7, 8 and 30 h, respectively (Figure 3d). The results thus confirmed that all polymer coatings decelerated the UCNP dissolution in PBS; the dissolution rate of UCNP@PMVEMA at 37 °C was lower (0.55 mol.%/h) than that of the UCNP@Ale-PEG (1.45 mol.%/h) and UCNP@Ale-PDMA particles (1.37 mol.%/h; Table 1). The molar fraction of Y^3+^ ion leakage (*X*_Y_ = 0.79 ± 0.24 mol.%) also suggested that PMVEMA protected the UCNPs from dissolution in PBS at 37 °C even after 72 h (Figure 3e). The highest dissolution in PBS was observed for the uncoated UCNPs, where *X*_Y_ reached 1.44 ± 0.01 mol.% under the same conditions. In the case of UCNP@Ale-PEG and UCNP@Ale-PDMA, leaching reached 1.19 ± 0.03 and 1.01 ± 0.20 mol.% of Y^3+^ ions, respectively (Figure 3e). To better understand how the UCNPs degrade under in vitro conditions, their dissolution was also studied in DMEM medium and ALF simulating the endosomal compartment at 37 °C. After 24 and 168 h of incubation in DMEM, the F^−^ leakage from the neat UCNPs, Ale-PEG-coated UCNPs, Ale-PDMA-coated UCNPs, and PMVEMA-coated UCNPs reached 5.3, 6.5, 5.1, 5 and 6.3, 8.1, 6.3, 6.2 mol.%, respectively (Figure S6a). In contrast to DMEM, the dissolution of all the UCNPs in ALF for 24 and 168 h was more pronounced, reaching *X*_F_ ~14 and ~20 mol.%, respectively (Figure S6b). Thus, the disintegration of the UCNPs was substantially faster in ALF than in DMEM. This difference can be explained by the formation of a protective protein corona in DMEM, which inhibited the dissolution of the particles [14]. In addition, various components of FBS-containing DMEM, such as albumin, lipoproteins, glycoproteins, and globulins, coated the particles and protected them from dissolution. Therefore, small differences were observed between the dissolution of the coated and uncoated particles in DMEM. Thus, it can be assumed that the UCNPs primarily dissolved in cells and not in the DMEM medium, as documented below in in vitro experiments where the toxicity of the UCNPs depended on the number of particles internalized by the cells.

The above results demonstrated that the PMVEMA-modified UCNPs with the highest content of coating provided the best protection against the particle degradation in PBS; however, they also had the highest F^−^ release in water among all the particles. This indicated that the amount of polymer on the particles was not the only parameter affecting the UCNP degradation, but also the coating chemistry, ζ-potential, and number of binding sites on the particle surface available for the attachment of polymer played a role. As mentioned above, the dissolution of the UCNPs was faster in PBS than in water due to the reaction of lanthanoids with negatively charged phosphate ions. In contrast to the neutral or positively charged UCNP@Ale-PEG and UCNP@Ale-PDMA particles, the negatively charged PMVEMA shell hindered the diffusion of phosphate ions to the particle surface by Coulomb repulsions, slowing down the particle disintegration. Compared to Ale-PEG and Ale-PDMA with only one phosphonate anchoring group per chain to graft the polymer, PMVEMA contained plenty of carboxyl groups, potentially forming many binding sites for yttrium and/or lanthanoids on the particle surface. As a result, long-term DLS measurements of UCNP@PMVEMA did not show any change in *D*_h_ and ζ-potential, proving good protection of PMVEMA against dissolution of the UCNPs in PBS. The multiple anchoring carboxyl groups of PMVEMA obviously sterically hindered the reaction with phosphates, therefore suppressing the UCNP dissolution, as described elsewhere, where the ligands with four phosphonate groups better protected the UCNPs against dissolution than those with only two phosphonate groups [42]. It can be thus concluded that the amount of hydrophilic polymer coating should be high enough, with multiple anchoring groups, and strong affinity to the metal ions, if the UCNPs with enhanced chemical and colloidal stability against degradation in PBS are to be designed.

### 2.5. Cytotoxicity by MTT Assay

**Figure 4:**
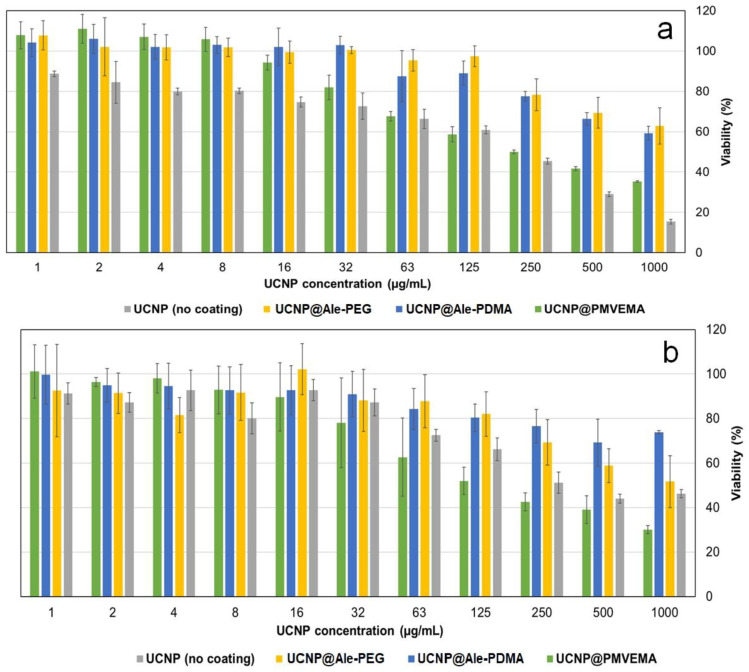
Dependence of viability of (**a**) C6 cells and (**b**) rMSCs on concentration of UCNPs, UCNP@Ale-PEG, UCNP@Ale-PDMA, and UCNP@PMVEMA after 1 day of incubation according to MTT assay.

Before prospective in vivo experiments on animals, it is necessary to test the cytoto-xicity of the UCNPs on cell cultures using MTT assay. Hence, two types of cells were selected, namely, commonly used C6 rat glioblastoma cells representing a model of tumor cells and rMSCs exemplifying healthy non-cancerous cells. In some cases, the viability reached >100%, which can be caused by inhomogeneous cell growth during the incubation. Among several factors affecting the cell viability and nanoparticle biocompatibility, their protective coating plays an important role. However, inside the cells, the protective coating can degrade, inducing cytotoxicity. The viability of the C6 cells incubated with lower concentrations of the UCNP@Ale-PEG and UCNP@Ale-PDMA particles (<125 µg/mL) for one day was high (~85%; Figure 4a); generally, viability ≥80% is considered to be nontoxic [44]. The viability of the C6 cells incubated with UCNP@PMVEMA and the neat UCNPs under the same conditions decreased to ~60%; this decrease continued with the additional increasing of particle concentration (Figure 4a). At UCNP@Ale-PEG and UCNP@Ale-PDMA concentration >125 µg/mL, the viability of the C6 cells ranged 60–80%. Similar results were also obtained for rMSCs incubated with the UCNP@Ale-PEG and UCNP@Ale-PDMA particles (125 µg/mL); the viability reached ~80% and it decreased to 55 and 70% after the incubation with UCNP@PMVEMA and neat UCNPs, respectively (Figure 4b). The viability further decreased at particle concentration >125 µg/mL. Further, other studies have also documented the effect of the surface coating, UCNP concentration, incubation time, and cell type on cytotoxicity [45,46,47]. For example, the aminosilica-functionalized UCNPs (0–200 µg/mL), with a thick silica shell incubated with RAW264.7 macrophages for 24 h, exhibited a higher viability than those with a thin shell [45]. The particle concentration of 12.5 µg/mL did not induce a substantial drop in viability; however, higher concentrations decreased the viability, depending on the type of coating and/or surface modification. Similarly, the incubation of rMSCs with polyethyleneimine (PEI)-coated UCNPs (100 μg/mL) for 24 h resulted in 60% viability [46]. Our results thus agreed with these findings, when the concentration range 0–600 µg/mL was tested by MTT assay for 24 h; no robust decline in the viability was observed up to concentrations of 16–32 µg/mL for any of the investigated coatings. In contrast, human dermal fibroblasts and HaCaT keratinocytes were viable, even in the presence of a particle concentration of 125 µg/mL [47].

### 2.6. Uptake of UCNPs by Cells

**Figure 5:**
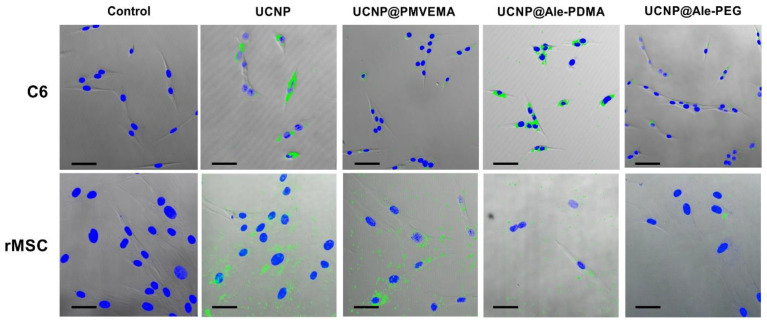
Light micrographs of rMSCs and C6 cells in the absence of UCNPs (control) and after 1-day incubation with UCNPs, UCNP@PMVEMA, UCNP@Ale-PDMA, and UCNP@Ale-PEG particles. Brightfield images were overlaid with confocal ones. Nanoparticles in cell cytoplasm were green and cell nuclei were blue (stained with DAPI). The highest uptake was seen with neat UCNPs, followed by UCNP@Ale-PDMA and UCNP@PMVEMA. The visualization of nanoparticles inside the cells corresponded to the results obtained from ICP-MS. Scale bar 50 µm.

The uptake of nanoparticles is important for future biomedical applications and cell viability. The uptake of all types of UCNPs by the rMSCs and C6 cells after 1-day incubation was visualized in a multiphoton laser scanning microscope. The largest number of particles was found in cells incubated with the neat UCNPs, followed by the UCNP@Ale-PDMA nanoparticles. In contrast, only a few UCNP@Ale-PEG particles were internalized by the cells (Figure 5).

**Figure 6:**
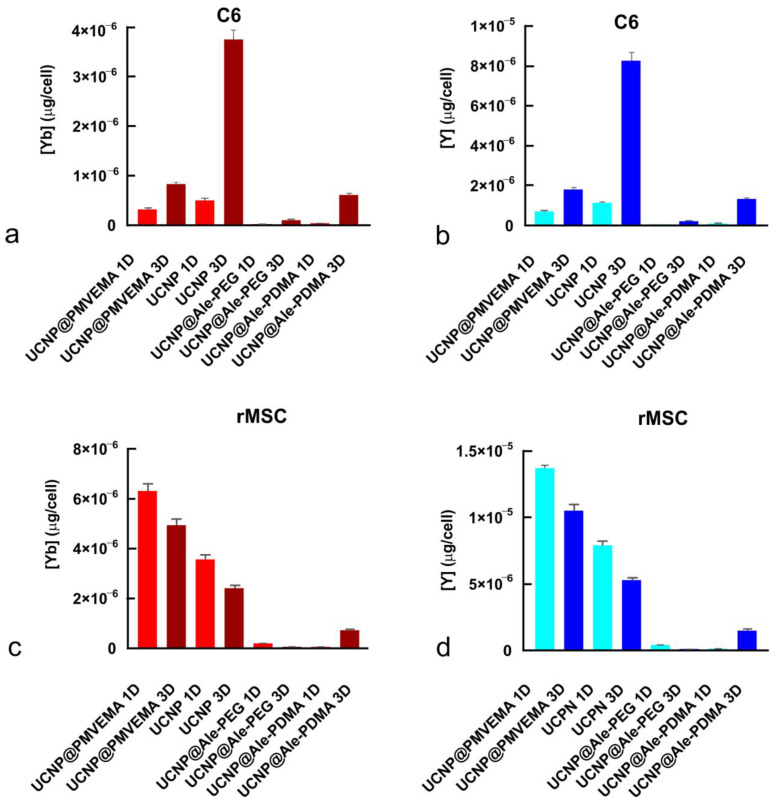
Amount of (**a**,**c**) Yb and (**b**,**d**) Y in (**a**,**b**) C6 cells and (**c**,**d**) rMSCs incubated with differently coated UCNPs for 1 or 3 days (denoted as 1D or 3D) according to ICP-MS.

To quantify the cellular uptake, the rMSCs and C6 cells were incubated with the UCNPs for 1 and 3 days and the content of Y and Yb was determined by ICP-MS. The highest concentration of Yb and Y (0.5 and 1.1 pg/cell, respectively) was found in the C6 cells incubated with neat UCNPs for 1 day. In the case of UCNP@PMVEMA, UCNP@Ale-PDMA, and UCNP@Ale-PEG, the concentrations of Yb and Y in the C6 cells were 0.3 and 0.7, 0.03 and 0.06, and 0.008 and 0.01 pg/cell, respectively. The prolonged incubation of the C6 cells with the UCNPs for 3 days further increased the Yb and Y concentrations inside the cells, up to 3.7 and 8.2 pg/cell for the neat UCNPs, 0.8 and 1.7 pg/cell for UCNP@PMVEMA, 0.6 and 1.3 pg/cell for UCNP@Ale-PDMA, and 0.09 and 0.2 pg/cell for UCNP@Ale-PEG, respectively (Figure 6 a,b). In rMSCs, the highest Yb and Y concentrations were found after 1-day incubation with UCNP@PMVEMA (6.3 and 13.7 pg/cell, respectively), which was followed by the neat UCNPs (3.6 and 7.9 pg/cell, respectively), UCNP@Ale-PDMA (0.06 and 0.14 pg/cell, respectively), and UCNP@Ale-PEG (0.21 and 0.43 pg/cell, respectively). In contrast to the C6 tumor cells where the Yb and Y concentration increased with increasing the incubation time, a longer incubation time (3 days) of rMSCs with the particles decreased the concentrations of Yb and Y in cells and the UCNP uptake, except of UCNP@Ale-PDMA. The final Yb and Y concentrations in rMSCs after 3 days of incubation with UCNPs, UCNP@PMVEMA, UCNP@Ale-PDMA, and UCNP@Ale-PEG were 2.4 and 5.3, 4.9 and 10, 0.74 and 1.5, and 0.07 and 0.1 pg/cell, respectively (Figure 6c,d).

The results thus confirmed that the C6 tumor cells were more sensitive to the pre-sence of the UCNPs than the rMSCs. This can be explained by steady internalization of the particles by the tumor cells during all three days, in contrast to rMSCs, the engulfment of which decreased after one day. Relative to the cell volume, the content of Y and Yb was higher in the C6 cells than in rMSCs, since the former cells were much smaller than the latter ones, which were closer to confluence as reported in the literature [48]. Moreover, the cell lines tended to internalize more nanoparticles than the primary cultures [49]. It is also interesting to note that the maximum concentration of Y and Yb in both cell types was similar. In addition, the extra- (pHe <6.8–7.1) and intracellular pH (pHi) in the tumor cells was more acidic and neutral (even alkaline), respectively, than that in the normal cells [50]. The abnormally high pHe/pHi ratio in the tumor cells is due to the high rate of glycolysis, which produces some acidic products (H_2_CO_3_ and CO_2_). The regulation of pHe/pHi ratio relies on several special proton pumps on the tumor cell membranes, such as SLC9A1 and V-ATPase [50]. As the particles can dissolve more in PBS (pH 7.4) than in water, it is possible that the UCNPs dissolve more in tumor cells than in rMSCs. The lowest cellular uptake was detected for the UCNP@Ale-PEG particles, as PEG is well-known to reduce non-specific interaction with cells [51]. At the same time, the UCNP@Ale-PEG particles were the least toxic, although they were less protected from degradation in PBS compared to UCNP@PMVEMA. Low cytotoxicity of UCNP@Ale-PEG thus may be due to low cellular uptake. On the other hand, the UCNP@PMVEMA particles exhibiting the highest cellular uptake induced the lowest cellular viability, while the UCNP@Ale-PDMA particles were relatively low toxic even at a relatively high uptake. In the literature, improved labeling efficiency of mouse MSCs was achieved with oligoarginine-conjugated PEG-coated UCNPs and the cytotoxicity was low even at a particle concentration of 200 µg/mL [52].

## 3. Materials and Methods

### 3.1. Materials

Octadec-1-ene (OD; 90%), ammonium fluoride (99.99%), anhydrous yttrium(III) and ytterbium(III) chlorides (99%), erbium(III) chloride hexahydrate (99%), *N*,*N*-dimethylacrylamide (DMA; 99%), 2,2′-azobis(2-isobutyronitrile) (AIBN), 4,4′-azobis(4-cyanovaleric acid) (ACVA), 2-(dodecylthiocarbonothioylthio)-2-methylpropionic acid (chain-transfer agent — CTA; 98%), 4-(dimethylamino)pyridine (DMAP; 99%), *N*,*N*′-dicyclohe-xylcarbodiimide (DCC; 99%), *N*-hydroxysuccinimide (NHS; 98%), and phosphate buf-fered saline (PBS) were obtained from Sigma-Aldrich (St Louis, MO, USA). Sodium salt trihydrate of (4-amino-1-hydroxy-1-phosphonobutyl)phosphonic acid (alendronate; Ale) was purchased from TCI (Tokyo, Japan). Methoxy poly(ethylene glycol) succinimidyl ester (PEG-NHS; *M*_w_ = 5 kg/mol) was purchased from Rapp Polymere (Tuebingen, Germany). 4′,6-Diamidino-2-phenylindole (DAPI) was from Thermo Fisher Scientific (Waltham, MA, USA). Poly(methyl vinyl ether-*co*-maleic acid) (PMVEMA; *M*_w_ = 60 kg/mol) was purchased from Scientific Polymer Products (Ontario, NY, USA). Oleic acid (OA; 98%), methanol (99.5%), hexane (99.5%), and dichloromethane (99.9%) were obtained from Lach-Ner (Neratovice, Czech Republic). *Tert*-butyl[2-(acryloylamino)ethyl]carbamate) (AEC-Boc) and PEG-alendronate (PEG-Ale) were synthetized according to the literature [53,54,55]. Suprapur^®^ nitric acid was purchased from Merck (Kenilworth, NJ, USA). Artificial lysosomal fluid (ALF; pH 4.5) was prepared according to previously published work [56]. All other reagent grade chemicals were purchased from Sigma-Aldrich and used as received. Cellulose dialysis membranes (MWCO 3.5 and 14 kg/mol) were purchased from Spectrum Europe (Breda, The Netherlands). ACVA was purified by recrystallization from methanol and the inhibitor was removed from DMA on a basic alumina column. Distilled demineralized water (conductivity ˂ 0.1 µS/cm) filtered on a Milli-Q Gradient A10 system (Millipore; Molsheim, France) was used throughout the experimental work.

### 3.2. Preparation of Poly(N,N-dimethylacrylamide-co-tert-butyl[2-(acryloylamino)ethyl]carbamate) [P(DMA-AEC-Boc)]

Statistical P(DMA-AEC-Boc) copolymer containing 5 mol.% of AEC-Boc was synthetized by a reversible addition-fragmentation chain-transfer (RAFT) polymerization. A 10-mL flask equipped with a magnetic stirrer was charged with DMA (1.2 g; 12.1 mmol), AEC-Boc (14 mg; 0.065 mmol), ACVA (9.8 mg; 0.035 mmol), CTA (56 mg; 0.154 mmol), and ethanol (2.9 mL), and sealed with a rubber septum. The mixture was purged with Ar for 20 min and polymerized at 70 °C for 30 min. After exposure to air and cooling in ice, the polymer was purified by repeated precipitation in hexane. CTA-end groups were removed by refluxing the methanolic solution (20 mL) of copolymer (0.3 g) with AIBN (50 mg) for 2 h and the resulting P(DMA-AEC-Boc) was purified by gel filtration in methanol on a Sephadex LH-10 column.

### 3.3. Modification of P(DMA-AEC) with Alendronate

Initially, carboxyl end groups of P(DMA-AEC-Boc) were activated via carbodiimide chemistry with NHS. A 20-mL flask equipped with a magnetic stirrer was loaded with NHS (10.35 mg; 0.09 mmol), DMAP (0.3 mg; 2.5 µmol), DCC (18.5 mg; 0.09 mmol), and acetone (8 mL), and the mixture was cooled to 5 °C in an ice bath. P(DMA-AEC-Boc) (200 mg; 0.018 mmol) was added and the reaction mixture was stirred at 5 °C for 4 h and at room temperature (RT) for 12 h. Precipitated dicyclohexylurea was removed by filtration with a Millex-HA syringe filter (0.45 μm pore size) and acetone was evaporated on a rotary evaporator at RT. In a 10-mL flask, NHS-activated P(DMA-AEC-Boc) (200 mg; 0.018 mmol) was added to 0.1 M PBS solution (5 mL; pH 7.4) of Ale (58.5 mg; 0.18 mmol) at 5 °C. After the dissolution, the mixture was vigorously stirred at RT for 48 h, dialyzed against water for 48 h (MWCO 3.5 kg/mol), and freeze-dried. Finally, the Boc-protecting groups were removed from P(DMA-AEC-Boc)-Ale (100 mg; 0.09 mmol) in 3 M methanolic HCl (3 mL) at RT for 2 h with stirring and methanol was removed at RT on a vacuum rotary evaporator. The resulting poly(*N*,*N*-dimethylacrylamide-*co*-2-aminoethylacrylamide)-alendronate [P(DMA-AEA)-Ale] was purified by dialysis against water for 48 h (MWCO 3.5 kg/mol) and lyophilized.

### 3.4. Synthesis of NaYF_4_:Yb, Er Nanoparticles (UCNPs)

UCNPs were prepared according to the earlier reported procedure [57]. Briefly, a 100-mL three-neck round-bottom flask equipped with a magnetic stirrer was loaded with YCl_3_ (0.78 mmol), YbCl_3_, (0.2 mmol), ErCl_3_∙6H_2_O (0.02 mmol), OA (6 mL), and OD (15 mL). The reaction mixture was stirred at 160 °C for 30 min under Ar atmosphere, the solution was cooled to RT, methanolic solutions (8 mL) of NH_4_F (148 mg; 4 mmol) and NaOH (100 mg; 2.5 mmol) were added, and the resulting dispersion was slowly heated to 120 °C to evaporate residual water and methanol under atmospheric pressure. After solvent evaporation, the reaction mixture was heated at 300 °C for 1.5 h. After cooling to RT, UCNPs were separated by centrifugation (3460 rcf) for 1 h, washed with hexane/ethanol mixture (1:1 *v*/*v*) twice, ethanol three times, ethanol/water (1:1 *v*/*v*) twice, and water twelve times (14 mL each), and dispersed in water.

### 3.5. Surface Modification of UCNPs with PEG-Ale, P(DMA-AEA)-Ale, and PMVEMA

In a 10-mL flask equipped with a magnetic stirrer, aqueous UCNP (30 mg) dispersion (1.64 mL) and aqueous solution (2 mL) of PEG-Ale or P(DMA-AEA)-Ale (15 mg) were loaded under sonication (Ultrasonic Homogenizer UP200S Hielscher; 20% power) for 1 min. The mixture was stirred at RT for 24 h. Polymer-modified UCNPs denoted as UCNP@Ale-PEG and UCNP@Ale-PDMA were purified via dialysis against water for 48 h (MWCO 14 kg/mol).

In the case of UCNP@PMVEMA, aqueous UCNP (15 mg) dispersion (1 mL) was added to aqueous PMVEMA solution (50 mg/mL; 15 mL; pH 7.4). The mixture was shaken for 30 min and continuously stirred at 70 °C for 16 h. The resulting particles were separated by centrifugation (14,100 rcf) and washed with water to remove unbound PMVEMA.

### 3.6. Characterization of UCNPs

The nanoparticle morphology was analyzed with a Tecnai Spirit G2 transmission electron microscope (TEM; FEI; Brno, Czech Republic) [38]. The number-average diameter (*D*_n_ = Σ N_i_∙*D*_i_/Σ N_i_), weight-average diameter (*D*_w_ = Σ N_i_∙*D*_i_^4^/Σ N_i_∙*D*_i_^3^), and dispersity (*Ð* = *D*_w_/*D*_n_) were calculated by the measurement of at least 300 particles from TEM micrographs using the Atlas software (Tescan Digital Microscopy Imaging; Brno, Czech Republic); N_i_ is the number and *D*_i_ is the diameter of the ith particle. The TEM was coupled with energy-dispersive X-ray (EDX) analysis (EDAX detector; Mahwah, NJ, USA). The hydrodynamic diameter (*D*_h_), ζ-potential, and polydispersity (*PD*) of nanoparticles were determined by dynamic light scattering (DLS) using a ZSU 5700 Zetasizer Ultra instrument (Malvern Instruments; Malvern, UK). ^1^H and ^31^P NMR spectra were recorded with a Bruker Avance III 600 spectrometer (Bruker; Billerica, MA, USA). Molar masses *M*_w_, *M*_n_, and *M*_w_/*M*_n_ of the polymers were determined by size exclusion chromatography (SEC) on a Shimadzu HPLC system (Tokyo, Japan) equipped with a UV-Vis diode array and OptilabrEX refractive index and DAWN EOS multiangle light scattering detectors (Wyatt; Santa Barbara, CA, USA). Infrared spectra were recorded on a 100T FTIR spectrometer (Perkin–Elmer; Waltham, MA, USA) using a Specac MKII Golden Gate single attenuated total reflection (ATR). Thermogravimetric analysis (TGA) was acquired with a Perkin–Elmer TGA 7 analyzer (Norwalk, CT, USA) over the temperature range 30–650 °C at a constant heating rate of 10 °C/min under air. The upconversion (UC) luminescence spectra of aqueous particle dispersions (4 mg/mL) were measured using a FS5 spectrofluorometer (Edinburgh Instruments; Edinburgh, UK) coupled with CW 980 nm infrared diode lasers as an excitation source with a nominal laser power of 2 W (MDL-III-980; beam size of 5 × 8 mm^2^).

### 3.7. Dissolution of UCNPs

In vials equipped with rubber septa, neat or coated UCNPs (1 mg/mL) were dispersed under stirring (250 rpm) in 0.01 M PBS (pH 7.4), water, DMEM or ALF at 25 or 37 °C for the selected time. The concentration of dissolved F^−^ ions in medium was determined using a combination fluoride electrode (Thermo Fisher Scientific; Waltham, MA, USA) according to the manufacturer’s instructions. Prior to the measurement, the particle dispersions were centrifuged (28,258 rcf) for 30 min to remove the majority of UCNPs and the supernatants were filtered (MWCO 30 kg/mol) to eliminate any residual UCNPs from the solution.

The leaching of Y^3+^ ions from the particles was determined using xylenol orange according to the previous report [58]. Briefly, nanoparticle dispersions (1 mg/mL) in PBS (4 mL) were stored at 37 °C for 24–72 h and the supernatants were prepared as described above. The solutions (0.2 mL) were then mixed with buffered xylenol orange (2 mL; pH 5.8) and the Y^3+^ concentration was monitored by a Specord 250 Plus UV-Vis spectrophotometer (Analytik; Jena, Germany) at 350–650 nm. The concentration of Y^3+^ ions released from UCNPs was directly proportional to the ratio of absorbances at 570 and 443 nm determined from the calibration curve of 18 μM xylenol orange in acetate buffer (pH 5.8) containing different amounts of YCl_3_ (0–70 μM of Y^3+^).

### 3.8. Cell Cultures

The C6 cells (kindly provided by Dr. Čestmír Altaner, Biomedical Research Center SAS, Bratislava, Slovak Republic) were thawed, washed in cold PBS, and plated in Dulbecco’s modified Eagle’s medium (DMEM; Thermo Fischer Scientific) containing fetal bovine serum (FBS; Merck; Darmstadt, Germany), primocin, and penicillin streptomycin (Gibco, Life Technologies; Grand Island, NY, USA) at 37 °C under 5% CO_2_ atmosphere. Primocin was added to the medium because it is a broad-spectrum antibiotic effective against mycoplasmas, for which the combination of penicillin and streptomycin is not sufficient. Moreover, primocin is also gentle on cell lines and is widely used. The medium was changed twice a week.

Rat mesenchymal stem cells (rMSCs) were isolated by aspirating bone marrow from the bones of two-month old rats (Velaz; Prague, Czech Republic). The rMSCs or C6 cells (both 1 mL) were thawed from liquid nitrogen in a dry bath incubator at 37 °C, fresh DMEM medium (9 mL) was added to disperse cells, and the suspension was centrifuged (3 rcf) for 10 min. After removal of the supernatant, the pellet was resuspended in DMEM medium (10 mL; low glucose—1000 mg/L, with L-glutamine and sodium bicarbonate), the cells were plated in a 75 cm^2^ culture flask, and incubated (PHCbi CO_2_ incubator; Tokyo, Japan) at 37 °C for 3 days. After the incubation, the cells were passaged, the DMEM medium was withdrawn, and the cells were washed with PBS and removed from the flask using 0.25% trypsin-ethylenediaminetetraacetic acid solution, centrifuged (3 rcf) for 10 min, and counted in Bürker chamber.

### 3.9. MTT Assay

The MTT test (MilliporeSigma; Burlington, MA, USA) was performed on three 96-well plates (Thermo Fischer Scientific) with each well containing a suspension (50 µL) of 10,000 C6 and/or rMSC cells per cm^2^. The cell suspensions were incubated at 37 °C for 24 h under 5% CO_2_ atmosphere, 50 µL of particle dispersions (0, 1, 2, 4, 8, 16, 32, 63, 125, 250, 500, and 1000 µg/mL) was added, and the incubation continued for another 24 h. Subsequently, the MTT solution (10 µL; 5 mg/mL) was added to each well, the mixture was incubated for another 4 h, 10% sodium dodecyl sulfate in 0.01 M HCl (100 µL) was added, and the incubation continued for 24 h until dissolution of the formazan crystals. The absorbance was measured on a Spark^®^ multimode microplate reader (Tecan; Männedorf, Switzerland) at 590 nm. The experiments were performed in triplicate.

### 3.10. Inductively Coupled Plasma Mass Spectrometry (ICP-MS)

A NexION 350D ICP-MS instrument (PerkinElmer; Woodbridge, ON, Canada), equipped with Universal Cell Technology™ for spectral interference elimination, was used for ICP-MS measurements. The sample introduction system included an internal peristaltic pump with Tygon^®^ tubing (0.38 mm internal diameter), polytetrafluoroethylene concentric nebuliser, and 100-mL glass cyclonic spray chamber.

The rMSCs and C6 cells were treated with concentrated nitric acid (3 mL) and transferred into Teflon vessels for microwave decomposition (Speedwave4; Berghof, Germany). The decomposed samples were transferred into a volumetric flask (50 mL) and after appropriate dilution spiked with the internal standard solution (^100^Rh). The calibration and internal standard solutions were prepared from the starting concentration of 1.000 ± 0.002 g/L (Merck, Darmstadt, Germany).

### 3.11. Confocal Microscopy

An Olympus FV1200 MPE multiphoton laser scanning microscope monitored the presence of nanoparticles inside the rMSCs and C6 cells. Briefly, the cells were fixed with 4% paraformaldehyde in PBS and stained with DAPI. Subsequently, brightfield images were taken at 980-nm excitation and 540-nm emission using an IR pulsed laser with negative chirp for multiphoton excitation. The DAPI-stained cells were visible in blue channel with laser diode at 405 nm.

## 4. Conclusions

The effect of a protective coating based on Ale-PEG, Ale-PDMA, and PMVEMA was investigated on the dissolution of spherical NaYF_4_:Yb^3+^,Er^3+^ UCNPs in PBS and water at 25 and 37 °C by potentiometry. The time-dependent concentration of F^−^ ions was higher in PBS than in water, which was related to the chemical structure of the polymer coating. All coatings suppressed the UCNP dissolution in PBS at both studied temperatures. Ale-PEG and Ale-PDMA decreased the dissolution of the UCNPs in water regardless of temperature, thus increasing biocompatibility and rendering the particles colloidally stable due to Ale groups that anchored the polymers to the particle surface. On the other hand, negatively charged PMVEMA slightly increased the UCNP dissolution in water but did not compromise the colloidal stability. In PBS, the UCNP@PMVEMA particles were less susceptible to dissolution and aggregation than the other nanoparticles, probably due to the multiplicity of the anchoring groups. Generally, the number of released ions was not necessarily decisive for inducing the toxicity of particles, as the polymer coatings and particle-cell interactions may play a more important role. Although PMVEMA showed the best protection against degradation of the UCNPs in PBS, their toxicity was more pronounced than in the case of other surface-modified UCNPs. For example, the PEG-coated UCNPs did not affect cell viability; however, they were the least internalized of all the surface-engineered particles investigated. The MTT test revealed that the cancer C6 cells were more sensitive to the presence of the UCNPs than rMSCs. Moreover, PDMA was identified as the most promising coating for the UCNPs to be used in cell experiments because it provided reasonably high cellular uptake and relatively low cytotoxicity by protecting the particles from dissolution in PBS; in addition, it provided superior colloidal stability in water. The amino groups also made PDMA suitable for subsequent conjugation with biomolecules. These biocompatible PDMA-coated UCNPs may find applications for deep-penetrating NIR photodynamic cancer therapy and/or imaging of cancer cells in the development of new drug formulations, taking advantage of safe excretion from the living organism after the therapeutic task is accomplished. This study also emphasized the importance of combined cytotoxicity and degradation testing to fully evaluate the safety of the UCNPs intended for in vitro and in vivo applications.

## Figures and Tables

**Table 1:** Characterization of differently coated UCNPs and their dissolution rates in PBS calculated from linear parts of curves in Figure 3.

Particles	*D*_n_ (nm)	*Đ*	*D*_h_ (nm)	*PD*	ζ-Potential (mV)	Coating ^a^ (wt.%)	Dissolution Rate (mol.%/h)	
							25 °C	37 °C
UCNPs	25	1.01	101 ± 1	0.20	30 ± 5	2.2	0.59 ± 0.07	1.96 ± 0.09
UCNP@Ale-PEG	25	1.01	68 ± 0.5	0.16	8 ± 1	10.3	0.25 ± 0.01	1.45 ± 0.01
UCNP@Ale-PDMA	25	1.01	102 ± 1	0.10	27 ± 3	7.2	0.28 ± 0.01	1.37 ± 0.01
UCNP@PMVEMA	24	1.01	112 ± 0.5	0.10	−32 ± 2	31.5	0.09 ± 0.00	0.55 ± 0.02

UCNPs—upconverting nanoparticles (NaYF_4_:Yb,Er); Ale-PEG—poly(ethylene glycol)-alendronate; Ale-PDMA—poly(*N*,*N*-dimethylacrylamide)-alendronate; PMVEMA—poly(methyl vinyl ether-*co*-maleic acid); *D*_n_—number-average diameter (TEM); *Ð*—dispersity (TEM); *D*_h_—hydrodynamic diameter (DLS); *PD*—polydispersity (DLS); ^a^—according to TGA.

## Data Availability

These data are available with M.N.

## Supplementary Materials

The following supporting information can be downloaded at: https://www.mdpi.com/article/10.3390/ijms24032724/s1.
