# Supplementary material for: Chemical and Colloidal Stability of Polymer-Coated NaYF4:Yb,Er Nanoparticles in Aqueous Media and Viability of Cells: The Effect of a Protective Coating
Source: Int J Mol Sci. 2023 Feb 1;24(3):2724. doi: 10.3390/ijms24032724 (PMC9917078; doi:10.3390/ijms24032724)
Supplement: Supplementary file 1 [file ijms-24-02724-s001.zip › ijms-2143870-supplementary.pdf]

Supporting information

Chemical and Colloidal Stability of Polymer-Coated $NaYF_4$:Yb,Er Nanoparticles in Aqueous Media and Viability of Cells: The Effect of a Protective Coating

Mykhailo Nahorniak [1], Vitalii Patsula [1], Dana Mareková [2,3], Petr Matouš [4], Oleksandr Shapoval [1], Viktoriia Oleksa [1], Magda Vosmanská [5], Lucia Machová Urdzíková [2], Pavla Jendelová [2,3], Vít Herynek [4]
and Daniel Horák [1,*]

[1] Institute of Macromolecular Chemistry, Czech Academy of Sciences, Heyrovského nám. 2, 160 00 Prague, Czech Republic; nahorniak@imc.cas.cz (M.N.); patsula@imc.cas.cz (V.P.); shapoval@imc.cas.cz (O.S.); oleksa@imc.cas.cz (V.O.)
[2] Institute of Experimental Medicine, Czech Academy of Sciences, Vídeňská 1083, 142 20 Prague, Czech Republic; dana.marekova@iem.cas.cz (D.M.); lucia.machova@iem.cas.cz (L.M.U.); pavla.jendelova@iem.cas.cz (P.J.)
[3] Department of Neurosciences, Second Faculty of Medicine, Charles University, V Úvalu 84, 150 06 Prague, Czech Republic
[4] Center for Advanced Preclinical Imaging, First Faculty of Medicine, Charles University, Salmovská 3, 120 00, Prague, Czech Republic; petr.matous@lf1.cuni.cz (P.M.); vit.herynek@lf1.cuni.cz (V.H.)
[5] Department of Analytical Chemistry, University of Chemistry and Technology Prague, Technická 5, Prague 6, 166 28 Prague, Czech Republic; magda.vosmanska@vscht.cz
* Correspondence: horak@imc.cas.cz

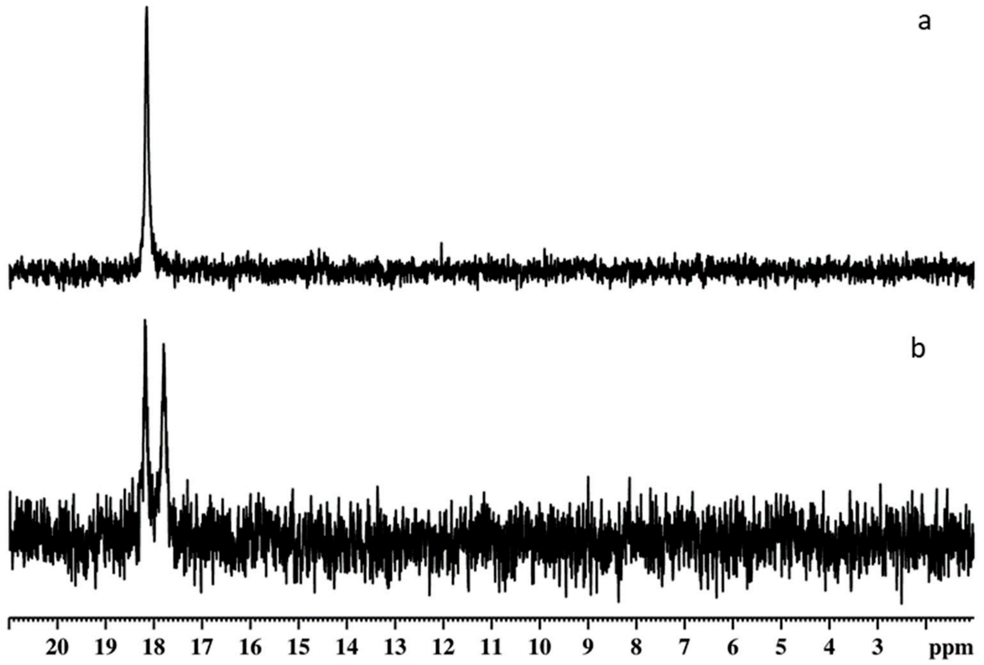

Figure S1. ^{31}P NMR spectra of (a) P(DMA-AEC-Boc)-Ale and (b) P(DMA-AEA)-Ale copolymers.

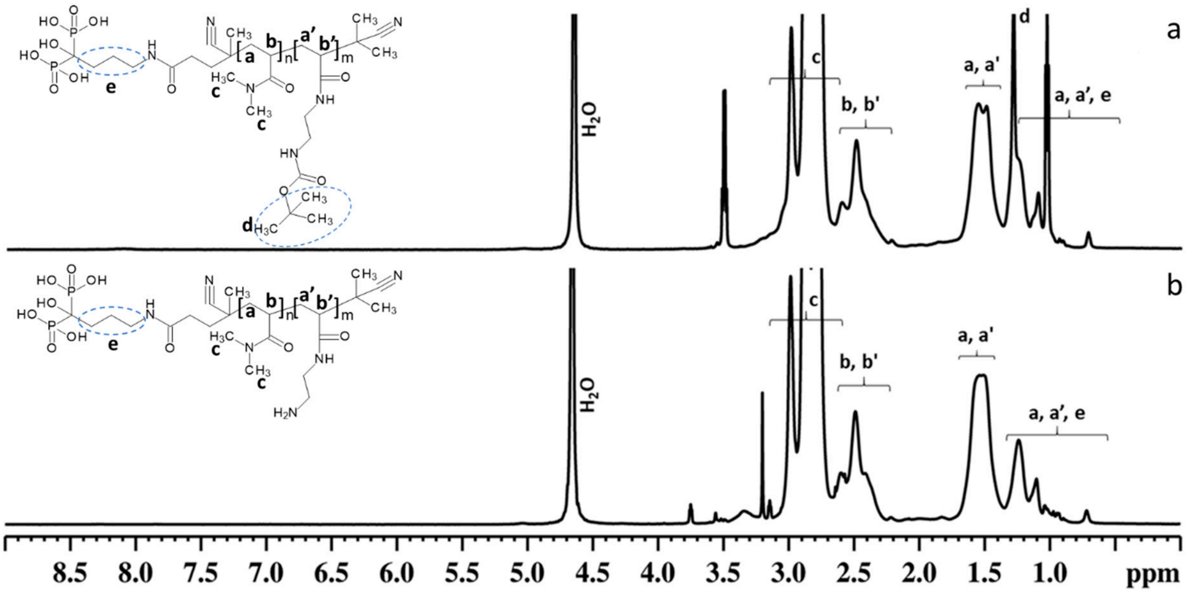

Figure S2. ^{1}H NMR spectra of (a) P(DMA-AEC-Boc) and (b) P(DMA-AEA)-Ale copolymers.

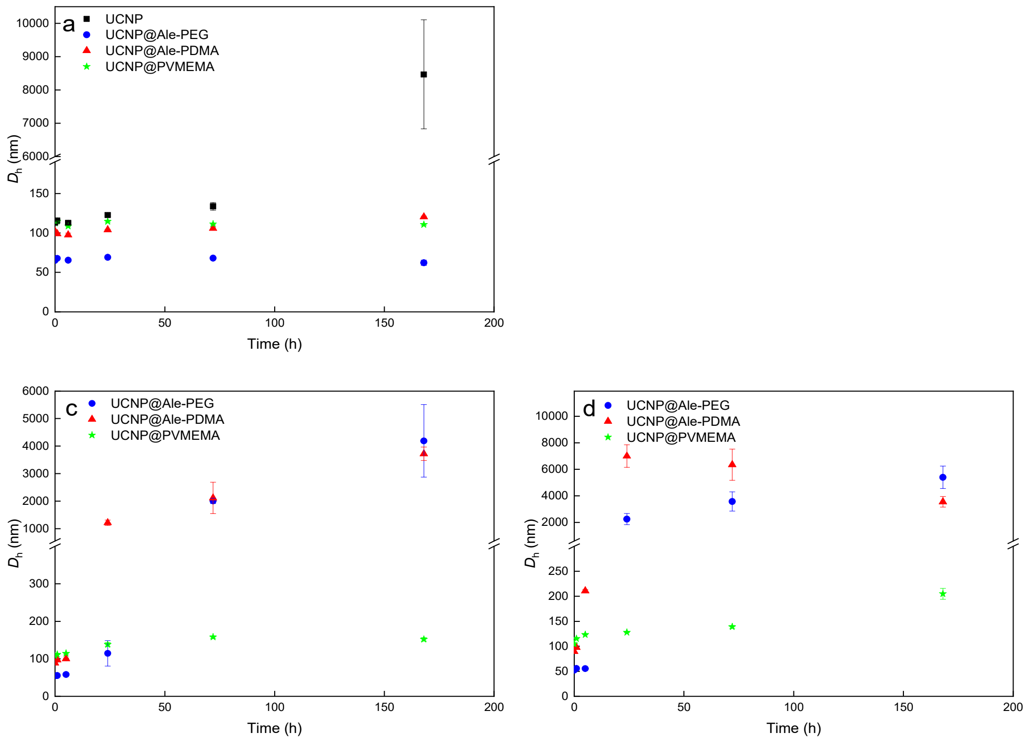

Figure S3. Time dependence of hydrodynamic diameter (D_h) of differently coated UCNPs in (a, b) water and (c, d) PBS (pH 7.4) at (a, c) 25 and (b, d) 37 °C.

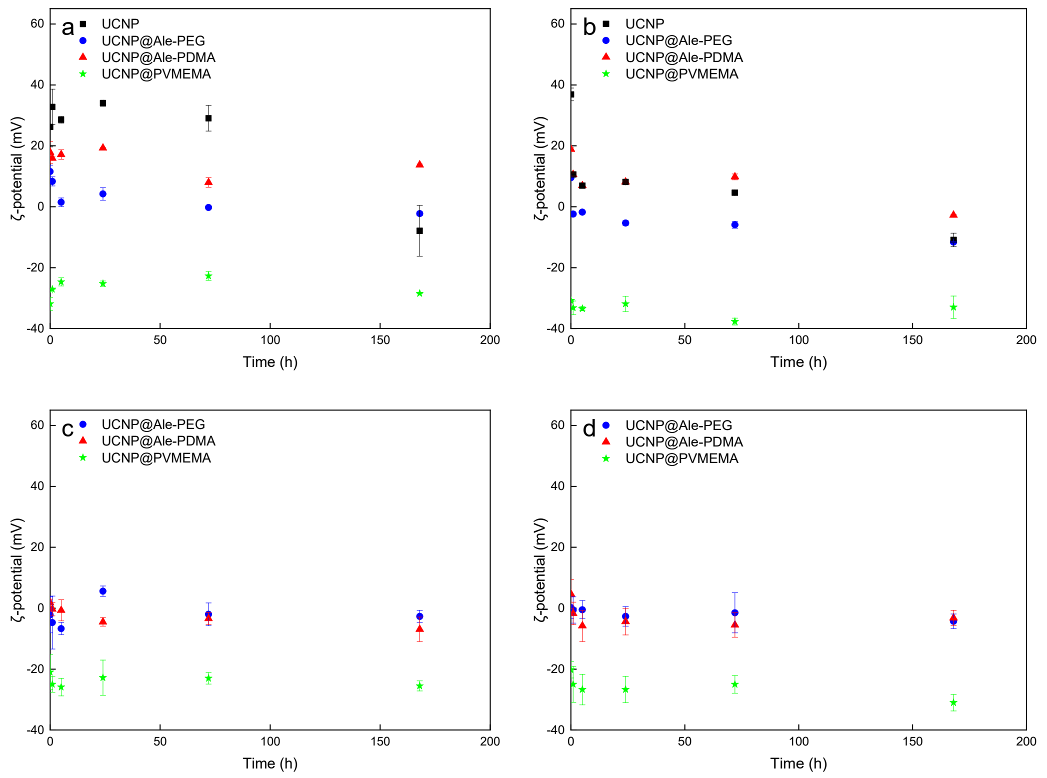

Figure S4. Time dependence of ζ-potential of differently coated UCNPs in (a, b) water and (c, d) PBS (pH 7.4) at (a, c) 25 and (b, d) 37 °C.

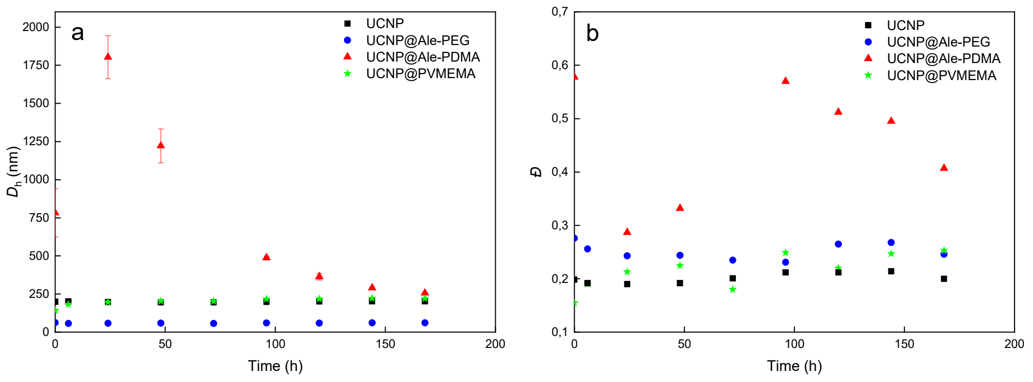

Figure S5. Time dependence of (a) hydrodynamic diameter D_h and (b) dispersity *Đ* of differently coated UCNPs in Dulbecco's modified Eagle's medium at 37 °C.

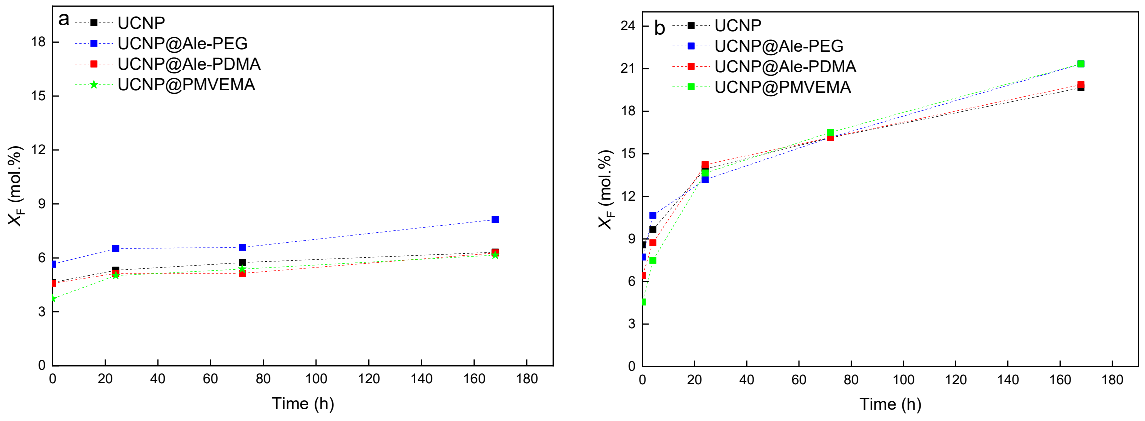

Figure S6. Time dependence of F⁻ molar fraction (X_F) of differently coated UCNPs in (a) Dulbecco's modified Eagle's medium and (b) artificial lysosomal fluid at 37 °C.
